# The PATH to Caregiving: Assessing Caregivers and Developing a Caregiver Plan of Care in the Acute Care Setting

**DOI:** 10.1093/geroni/igaa057.250

**Published:** 2020-12-16

**Authors:** Michelle Camicia, Barbara Lutz

**Affiliations:** 1 Kaiser Permanente, Novato, California, United States; 2 University of North Carolina-Wilmington, Wilmington, North Carolina, United States

## Abstract

Family caregivers of older adults report lack of preparation for their role, particularly upon acute hospital discharge following a medical event. Addressing the needs of family caregivers in the acute care setting prior to hospital discharge requires the identification of the caregiver, an assessment of caregiver preparedness, and a plan of care to address gaps in preparedness. The Preparedness Assessment for the Transition Home 7-item (PATH-7) is a valid and reliable instrument developed to assess family caregivers readiness for the caregiving role during acute care. The PATH-7 paper-pencil self-administered assessment was implemented in clinical care in medical-surgical nursing units in 2 acute care hospitals. Interventions to address gaps in preparedness were selected from a catalogue of interventions to develop a caregiver plan of care. The most frequent challenge identified by family caregivers was fulfilling the caregiving role on top of their other roles and responsibilities. This illustrated the need to assist family caregivers with exploring options for recruiting others to help with their roles and responsibilities and identify solutions soliciting and organizing help. This novel program promotes addressing the needs of the family unit, moving to a family-integrated are delivery model. Implementation challenges included in-person contact with caregiver to administer assessment, resources to respond to identified gaps in readiness, and lack of technology-enabled assessment administration. Positive staff experience with identifying and addressing needs of caregivers was a facilitator of staff engagement. Identifying, assessing, and addressing the needs of family caregivers of older adults is feasible in the acute care setting.

